# High-dose vitamin B1 therapy prevents the development of experimental fatty liver driven by overnutrition

**DOI:** 10.1242/dmm.048355

**Published:** 2021-03-18

**Authors:** Mugagga Kalyesubula, Ramgopal Mopuri, Jimmy Asiku, Alexander Rosov, Sara Yosefi, Nir Edery, Samuel Bocobza, Uzi Moallem, Hay Dvir

**Affiliations:** 1 Institute of Animal Science, Volcani Center - Agricultural Research Organization (ARO), Rishon LeZion 7528809, Israel; 2 Department of Animal Science, The Hebrew University of Jerusalem, Rehovot 7610001, Israel; 3 Institute of Biochemistry, Food Science and Nutrition, The Hebrew University of Jerusalem, Rehovot 7610001, Israel; 4 Pathology Laboratory, Kimron Veterinary Institute, Veterinary Services, Rishon LeZion 50250, Israel; 5 Institute of Plant Sciences, Volcani Center - ARO, Rishon LeZion 7528809, Israel

**Keywords:** Thiamine, Steatosis, Fatty liver, Obesity, Metabolic syndrome, NAFLD, Insulin resistance

## Abstract

Fatty liver is an abnormal metabolic condition of excess intrahepatic fat. This condition, referred to as hepatic steatosis, is tightly associated with chronic liver disease and systemic metabolic morbidity. The most prevalent form in humans, i.e. non-alcoholic fatty liver, generally develops due to overnutrition and sedentary lifestyle, and has as yet no approved drug therapy. Previously, we have developed a relevant large-animal model in which overnourished sheep raised on a high-calorie carbohydrate-rich diet develop hyperglycemia, hyperinsulinemia, insulin resistance, and hepatic steatosis. Here, we tested the hypothesis that treatment with thiamine (vitamin B1) can counter the development of hepatic steatosis driven by overnutrition. Remarkably, the thiamine-treated animals presented with completely normal levels of intrahepatic fat, despite consuming the same amount of liver-fattening diet. Thiamine treatment also decreased hyperglycemia and increased the glycogen content of the liver, but it did not improve insulin sensitivity, suggesting that steatosis can be addressed independently of targeting insulin resistance. Thiamine increased the catalytic capacity for hepatic oxidation of carbohydrates and fatty acids. However, at gene-expression levels, more-pronounced effects were observed on lipid-droplet formation and lipidation of very-low-density lipoprotein, suggesting that thiamine affects lipid metabolism not only through its known classic coenzyme roles. This discovery of the potent anti-steatotic effect of thiamine may prove clinically useful in managing fatty liver-related disorders.

This article has an associated First Person interview with the joint first authors of the paper.

## Abstract (precis)

**Editor's choice:** Experiments using overnourished sheep raised on a high-calorie liver-fattening diet, treated with and without thiamine, revealed that vitamin B1 protects against the development of fatty liver driven by overnutrition.

## INTRODUCTION

Fatty liver (FL) represents an abnormal metabolic condition of excess intrahepatic fat (>5.5% w/w liver fat fraction), which is also referred to as hepatic steatosis. FL and its association with chronic liver disease (steatohepatitis, cirrhosis and cancer) has long been known to result from excessive alcohol consumption or viral hepatitis (Asselah et al., 2006; Louvet and Mathurin, 2015). However, in recent decades the prevalence of non-alcoholic FL disease (NAFLD), which is largely attributed to excess caloric intake and sedentary lifestyle, has become more significant and is currently estimated to affect ∼25% of the global population (Younossi et al., 2018). To better reflect the tight relationship of NAFLD with systemic metabolic dysfunction, such as insulin resistance and dyslipidemia, and its continuum of liver abnormalities, its coexistence with other liver diseases or concomitant to alcohol consumption, an alternative name, i.e. metabolic dysfunction-associated FL disease (MAFLD), has recently been proposed (Eslam et al., 2020a,b). We have adopted this more-inclusive term in the following.

Despite the increasing prevalence of MAFLD, and the accompanying rise in end-stage liver disease requiring liver transplantation (Goldberg et al., 2017; Wong et al., 2015) and in hepatocellular carcinoma (Mittal et al., 2016), there is as yet no approved drug therapy for the disorder. Although numerous therapeutic strategies are being developed and tested for targeting the steatotic, oxidative, inflammatory, fibrotic and metabolic aspects of MAFLD, the responses observed in clinical trials have so far been inadequate (Friedman et al., 2018). This may reflect the heterogeneous and multifaceted nature of the disease, highlighting the unmet need for more robust preclinical models and for the development of combination therapies (Santhekadur et al., 2018). Likewise, therapeutic agents with the potential to counteract multiple aspects of this complex disorder may be of higher clinical value.

We have recently reported a large-animal nutritional model for FL, characterized by excessive carbohydrate intake, hyperglycemia and hyperinsulinemia, which collectively drove liver steatosis in sheep (Kalyesubula et al., 2020). In a search for a pharmacological intervention with the potential to counteract both the systemic and liver-related metabolic aspects of this model, we decided to investigate thiamine, also known as vitamin B1, as a plausible beneficial agent, because of its fundamental role in energy metabolism. The biologically active form of thiamine is produced by its intracellular phosphorylation to thiamine pyrophosphate (TPP) (Voskoboyev and Ostrovsky, 1982), an essential coenzyme for the catalytic decarboxylation of α-keto acids and transketolase reactions in all forms of life (Manzetti et al., 2014).

Of particular relevance to overnutrition and related metabolic disorders is the role of TPP in mitochondrial catabolism as a coenzyme for pyruvate dehydrogenase (PDH) and α-ketoglutarate dehydrogenase (α-KGDH). In the decarboxylation of pyruvate to acetyl coenzyme A (acetyl-CoA) by PDH, TPP enables the terminal oxidation of carbohydrate-carbon sources in the tricarboxylic acid (TCA) cycle. Whereas this role places thiamine bioavailability at a pivotal checkpoint between aerobic and anaerobic glucose metabolism, the cofactor role of TPP for α-KGDH, which is involved in the regulation of the turnover rate of the TCA cycle (Huang et al., 2003), emphasizes the importance of thiamine in the catabolism of both carbohydrates and fatty acids.

Regular ingestion of western-style diets, particularly rich in simple carbohydrates, can deplete physiological levels of thiamine (Elmadfa et al., 2001; Lonsdale, 2006; Maguire et al., 2018). Indeed, thiamine deficiency is associated with obesity and diabetes (Eshak and Arafa, 2018; Thornalley and Ali, 2007), and thiamine supplementation reduced hyperglycemia, diabetic complications and oxidative stress (Al-Attas et al., 2014; Alaei Shahmiri et al., 2013; Arvidsson et al., 2003; Babaei-Jadidi et al., 2004; Gorlova et al., 2019; Hammes et al., 2003; Karachalias et al., 2010; Rabbani et al., 2009; Tunc-Ozdemir et al., 2009). In the metabolic context, it has been shown that overproduction of thiamine in *Arabidopsis* plants increases the oxidation of carbohydrates in the TCA cycle (Bocobza et al., 2013). Taken together, these observations suggest that thiamine concentrations above the normal physiological levels may enhance carbohydrate combustion, presumably although not necessarily exclusively, by maximizing the oxidative activity of TPP-dependent enzymes. Whether such thiamine-dependent action can slow the rate of hepatic fat accumulation by reducing carbohydrate availability for *de novo* lipogenesis (DNL) and/or by directly increasing fatty acid oxidation in the TCA-cycle remains unexplored.

The strong association between hepatic steatosis and hyperglycemia in the sheep model (Kalyesubula et al., 2020), together with the accumulated evidence in respect to the relevant metabolic benefits of thiamine supplementation, motivated us to investigate the potential of high-dose thiamine treatment to counteract the overnutrition-driven steatosis that develops in this model.

## RESULTS

**Fig. 1.**
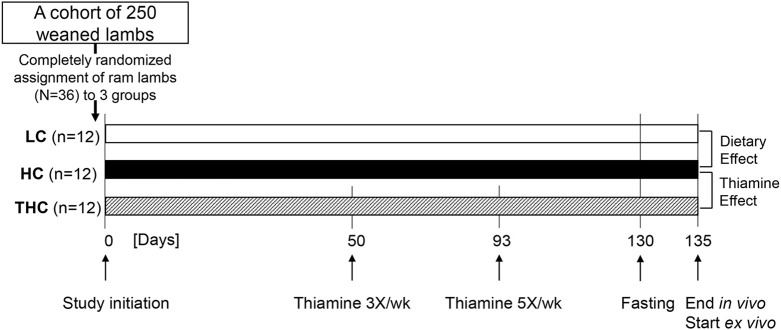
**Experimental design and timeline**.

To test the hypothesis that high-dose thiamine treatment can reduce hepatic steatosis, we conducted an experiment in which randomly assigned weaned lambs (*N*=36) were raised for a total of 135 days under three different regimes (Fig. 1). These were, (i) a low-calorie (LC) diet known to maintain lean livers, (ii) a high-calorie (HC) diet known to stimulate FL (Kalyesubula et al., 2020), and (iii) thiamine-treatment in combination with the HC diet (THC).

Subcutaneous injections of thiamine (300 mg/animal) were initiated on day 50, to allow for some liver fat to build up. Metabolic and physical parameters were weekly monitored *in vivo* throughout the entire experiment, and the cumulative liver phenotypes were evaluated *ex vivo* on livers harvested postmortem. Differences between HC and LC animals were ascribed to effects of the diet, whereas differences between THC and HC animals (from day 50 onwards) to the thiamine treatment effects.

To identify molecular signals associated with the observed phenotypes, we employed differential expression analyses of selected genes involved in carbohydrate and lipid catabolism, synthesis, transport and storage, since improper balance between these metabolic pathways may lead to abnormal accumulation of intrahepatic fat (Kawano and Cohen, 2013).

### High-calorie diet increased weight gain and adiposity

As expected (Kalyesubula et al., 2020), animals raised on the HC diet gained substantially more weight than those consuming the LC diet (*P<*0.0001; Table 1, Fig. S1). This increase in body weight was accompanied by significantly greater adiposity compared with animals on the LC diet, as indicated by a higher body mass index (BMI; *P=*0.003) and a higher body condition score (BCS; *P*<0.0001), determined on a scale of 1 to 5, ranging from very thin to very fat (Kenyon et al., 2014) (Table 2). On average, THC animals consumed similar or slightly higher quantities of the HC diet than HC animals (Table S1 and Fig. S2A) but tended to weigh less after the same number of days (*P*=0.09; Table 1, Table S2).

**Table 1.**
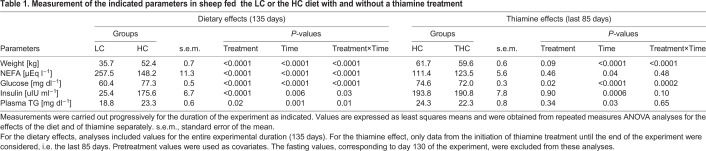
**Measurement of the indicated parameters in sheep fed the LC or the HC diet with and without a thiamine treatment**

**Table 2.**
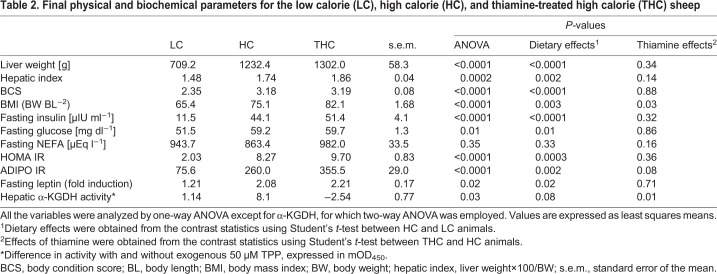
**Final physical and biochemical parameters for the low calorie (LC), high calorie (HC), and thiamine-treated high calorie (THC) sheep**

Consistent with adiposity induced by HC diet, HC animals presented with higher levels of fasting plasma leptin than LC animals (*P=*0.02; Table 2, Fig. S3A). The circulating concentrations of triglycerides (TG) were also significantly higher in HC compared with LC animals (*P=*0.02; Table 1, Fig. S3B). However, no effects of thiamine on either plasma leptin or TG were detected.

### Thiamine raised the liver abundance of TPP, as measured by α-KGDH activity

**Fig. 2.**
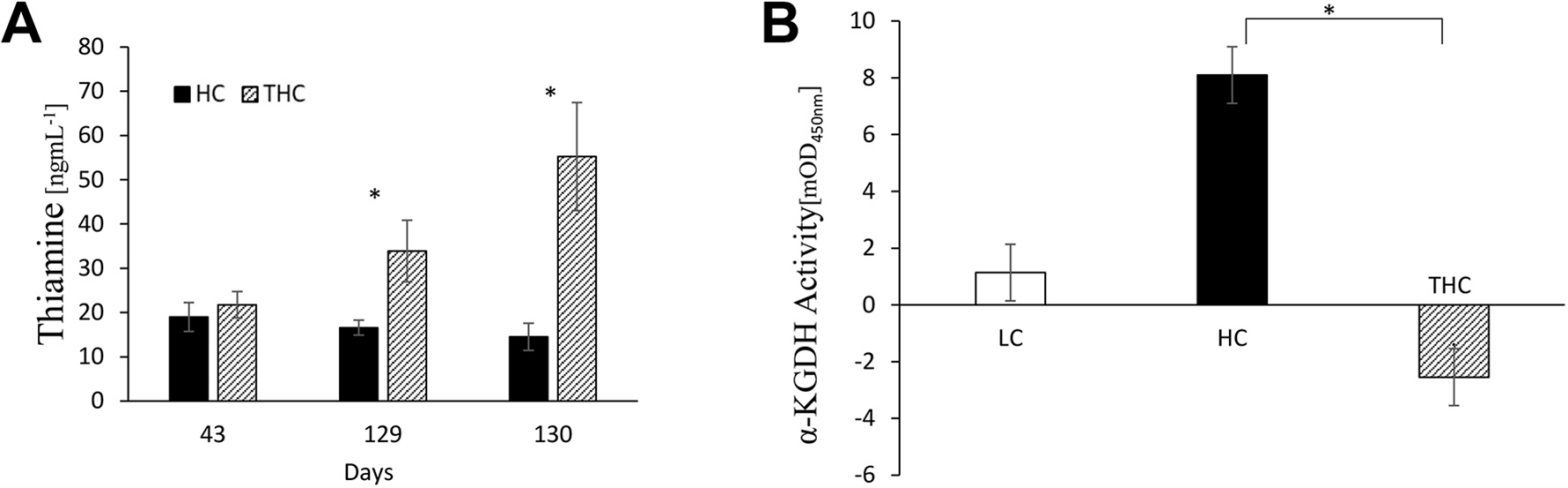
**Analyses of thiamine levels in plasma and activity of** α**-KGDH in liver in response to exogenous TPP in LC, HC** **or** **THC sheep.** (A) Concentrations of plasma thiamine before thiamine treatment (day 43) and towards the end of the experiment, i.e. days 129 and 130 (fasting) in HC and THC sheep. At each time point, data were analyzed by a Student's *t*-test. (B) Additive α-KGDH activity in liver, plotted in response to exogenous addition of 50 µm TPP in LC, HC and THC sheep. Plotted data represent the mean activity values averaged during 25 min from the initiation of the enzymatic reaction. Repeated measures ANOVA of activity measurements collected every 5 min for a total of 25 min detected a significant effect of treatment (*P=*0.03). *P<0.05 for the difference between HC and THC by contrast statistics using Student's *t*-tests, reflecting a relative hepatic TPP deficiency in the HC animals. Negative values for the THC group imply that endogenous levels of TPP are high and close to the maximum potential effect regarding α-KGDH activity, i.e. exogenous TPP had an inhibitory effect on α-KGDH activity. Error bars in A and B indicate the ±s.e.

As expected, thiamine-treated animals displayed higher circulating thiamine concentrations compared to the untreated HC group (*P=*0.05; Fig. 2A), indicating that the subcutaneous injections of thiamine hydrochloride did, indeed, reach the bloodstream efficiently. However, as this does not prove a concomitant increase of thiamine in tissue, the latter was evaluated through determination of intracellular TPP levels.

Generally, thiamine deficiency is diagnosed by evaluating the response of erythrocytes transketolase activity to exogenous TPP, where higher responses reflect higher deficiencies and vice versa (Mastrogiacomo et al., 1993). To examine the effect of the HC diet and the thiamine treatment on the abundance of TPP in the liver, we employed the same approach and studied the activity of α-KGDH in liver lysates in response to exogenous TPP. We found that α-KGDH activity increased more substantially in HC versus in THC animals in response to exogenous 50 µM TPP (*P=*0.01; Table 2). Although the response to TPP was not significantly different between THC and LC animals (*P*=0.3), the response in HC animals trended higher compared with that in LC animals (*P*=0.08). Taken together, these enzymatic data indicate that the HC diet induced low hepatic levels of TPP, and that thiamine treatment reversed this effect (Fig. 2B).

### Thiamine reduced blood glucose and increased liver glycogen levels

**Fig. 3.**
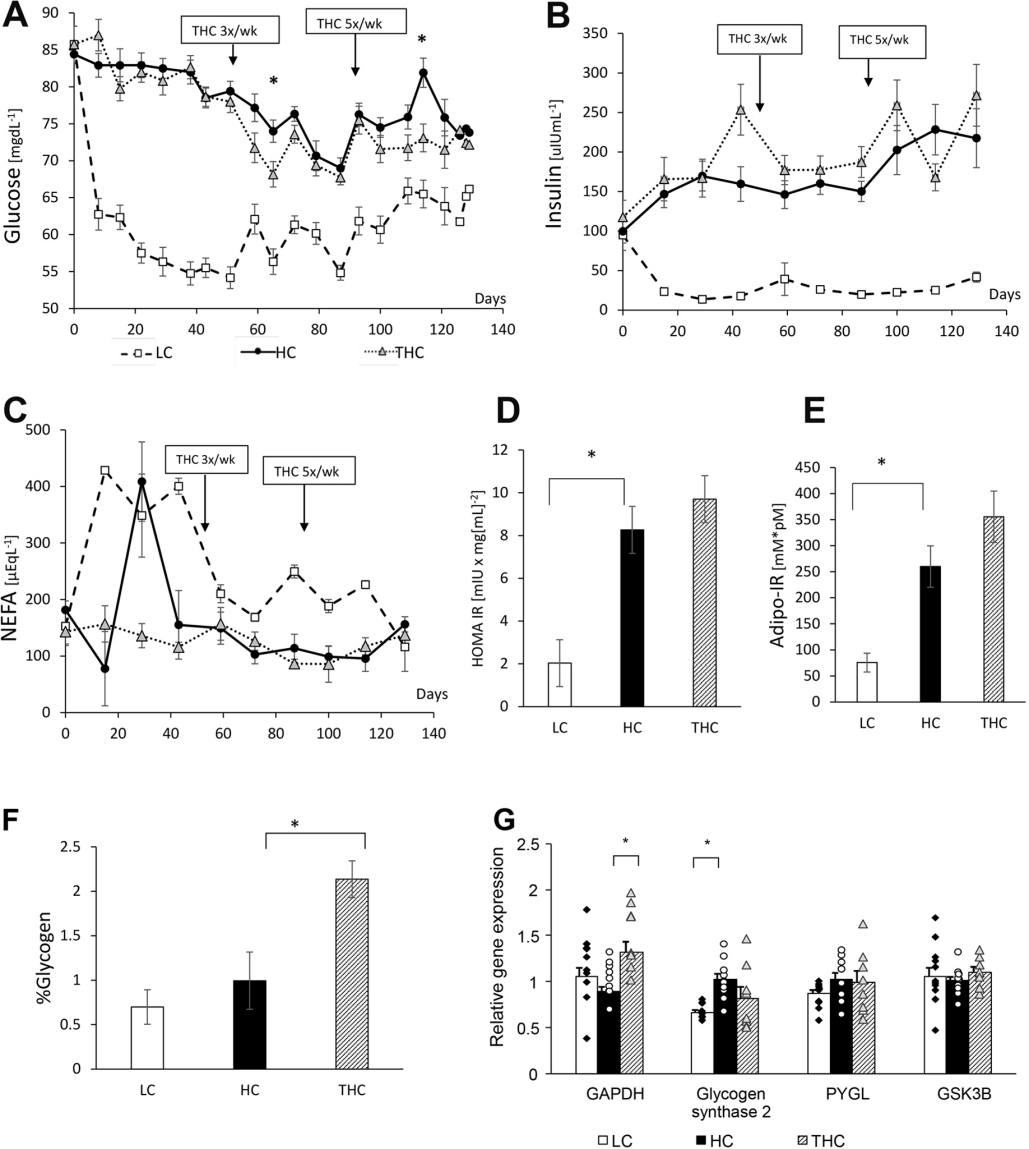
**Systemic and tissue metabolic responses in LC, HC and THC sheep.** (A) Levels of blood glucose. Data analysis for HC vs LC by repeated measures ANOVA revealed an effect of the dietary Treatment (*P*<0.0001), Time (*P*<0.0001) and Treatment×Time interaction (*P*<0.0001). Data analysis for HC vs THC revealed an effect of the thiamine Treatment (*P=*0.02), Time (*P<*0.0001) and Treatment×Time interaction (*P=*0.0002). *P<0.05 for the difference between HC and THC. (B) Levels of plasma insulin. Data analysis for HC vs LC revealed an effect of dietary Treatment (*P*<0.0001), Time (*P=*0.006) and Treatment×Time interaction (*P=*0.03). Data analysis for HC vs THC revealed no effect of treatment. (C) Levels of NEFA in plasma over time. Data analysis for HC vs LC revealed an effect of the dietary Treatment (*P*<0.0001), Time (*P*<0.0001) and Treatment×Time interaction (*P*<0.0001). Data analysis for HC vs THC revealed no effect of Treatment. (D) Whole-body insulin resistance as measured by HOMA-IR. One-way ANOVA detects an effect of Treatment (*P<*0.0001). (E) Adipose tissue insulin resistance as measured by ADIPO-IR. One-way ANOVA detects an effect of Treatment (*P<*0.0001). (F) Hepatic glycogen content. Data analysis by one-way ANOVA detected an effect of Treatment (*P=*0.001). (G) Expression of *GAPDH* and genes involved in glycogen metabolism. One-way ANOVA detected an effect of thiamine on *GAPDH* (*P=*0.01) and of the HC diet on glycogen synthase 2 (*P=*0.02) gene expression. *P<0.05 by contrast statistics using Student's *t*-tests. GSK3B, glycogen synthase kinase-3 beta PYGL, glycogen phosphorylase (liver form).

As previously observed (Kalyesubula et al., 2020), in contrast to the LC diet, the HC diet induced fed-state hyperglycemia (*P*<0.0001; Fig. 3A, Table 1). The difference in blood glucose between HC and LC animals during fasting was not as substantial but still statistically different (*P=*0.01; Table 2). Similarly, steady fed-state hyperinsulinemia was induced by the HC diet (*P<*0.0001; Fig. 3B). THC animals had reduced blood glucose concentrations compared to the thiamine-untreated HC animals (*P=*0.02; Fig. 3A, Table 1). Despite its reducing effect on blood glucose levels, thiamine had no detectable effect on the diet-induced hyperinsulinemia (Fig. 3B). Since the average dietary intake was similar, or even slightly higher, in the THC compared to the HC group (Fig. S2), these observations suggest that thiamine-treated animals had enhanced carbohydrate catabolism. Consistently, we found that THC animals had increased mRNA levels (*P*=0.002; Fig. 3G) of the significant glycolytic factor, glyceraldehyde 3-phosphate dehydrogenase (*GAPDH*).

In line with the high plasma insulin levels observed in both HC and THC animals, the concentration of circulating non-esterified fatty acids (NEFA) were low in these groups compared with the LC group (*P*<0.0001; Fig. 3C, Table 1), indicative of reduced adipose lipolysis, presumably caused by the high plasma insulin. As previously observed (Kalyesubula et al., 2020), both whole-body and adipose-tissue insulin resistance were increased with the HC diet, as respectively evaluated by homeostatic model assessment of insulin resistance (HOMA-IR; *P*<0.0001) and ADIPO-IR (*P*<0.0001). However, neither were affected by the thiamine treatment (Fig. 3D,E, Table 2).

Interestingly, although blood glucose levels were lowered by thiamine, hepatic glycogen levels increased (Fig. 3F). Since thiamine did not affect plasma insulin, which together with glucose as a substrate for glycogen, is known to upregulate hepatic glycogen levels (Samuel and Shulman, 2016), the increase in hepatic glycogen by thiamine might indicate improved hepatic sensitivity to insulin. At gene expression levels, no differences were detected between THC and HC animals in genes related to insulin resistance (Fig. 3D,E, Fig. S4) or to glycogen formation (Fig. 3G), suggesting that post-translational effects that are known to regulate hepatic glycogen metabolism (Roach et al., 2012) are involved.

### Thiamine treatment prevented diet-induced hepatic steatosis

**Fig. 4.**
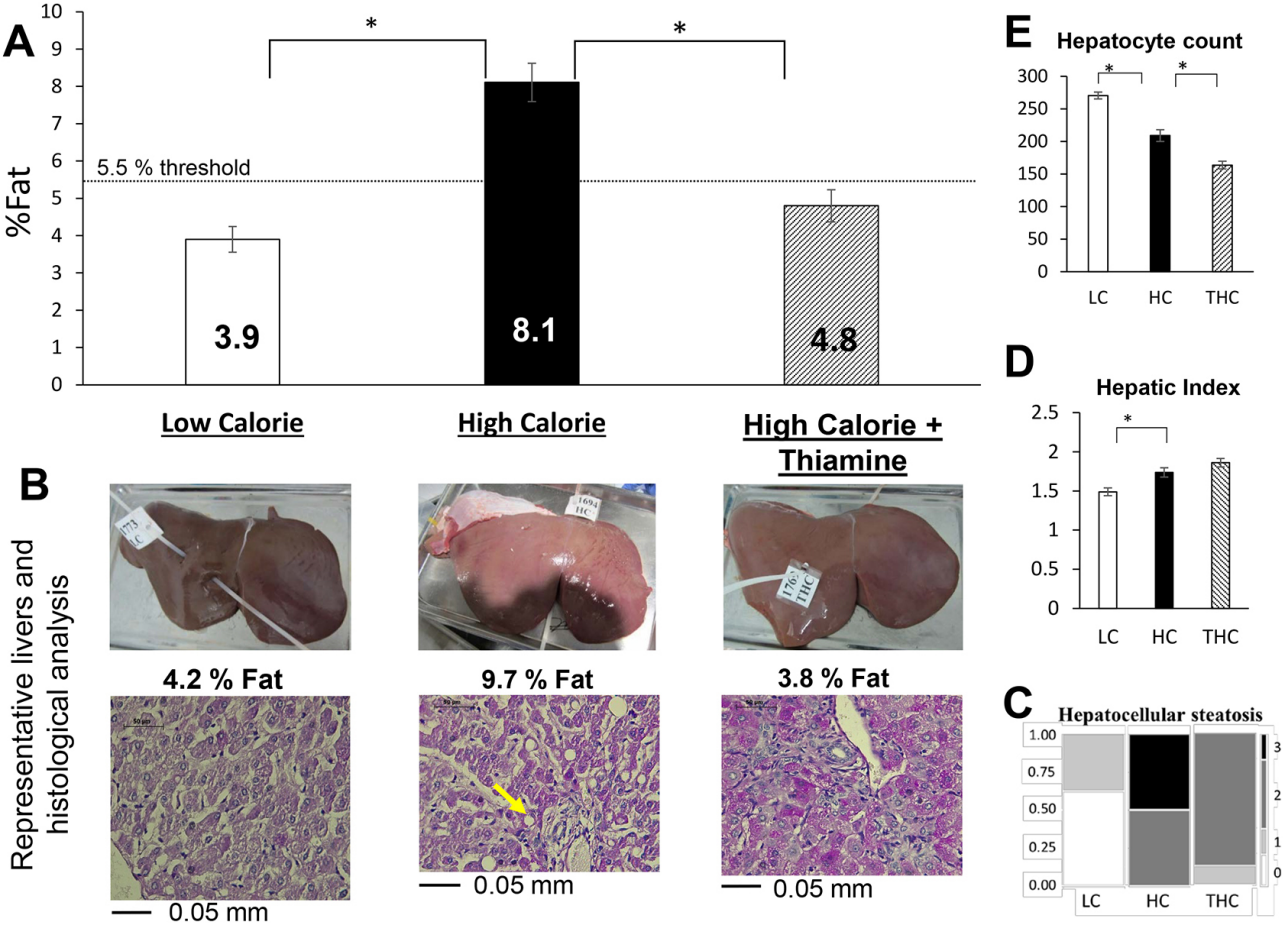
**Liver response** **in** **LC, HC and THC** **sheep.** (A) Levels of hepatic fat content. The HC diet promoted significant hepatic fat accumulation that was ameliorated by thiamine treatment (one-way ANOVA, *P*<0.0001). Similarly, contrast statistics using Student's *t*-tests between HC and LC, as well as between HC and THC, were highly significant (*P*<0.0001). (B) Representative images of livers and corresponding tissue sections that were analyzed by histopathology with H&E staining, taken at a ×200 magnification. Although macrovesicular steatosis was observed in HC lambs, microvesicular steatosis was more consistently present, mainly around lobular zone 3, and occasionally in zone 1. The arrow indicates macrovesicular steatosis where the hepatocyte nucleus was displaced to the side by a large lipid droplet. (C) Mosaic frequency plot of hepatocellular steatosis (scored for the presence of macrovesicular or microvesicular steatosis) graded between 0 and 3 (Materials and Methods). Differences between treatments were significant for both the Pearson and the likelihood ratio χ squares (*P*<0.0001). (D) Hepatic index values show the liver weight per animal body weight in percent. One-way ANOVA detected an effect of treatment (*P=*0.0002). (E) Quantification of the hepatocyte count analyzed under a fixed microscopic field at ×200 magnification. A high count reflects smaller hepatocytes and vice versa. *P<0.05 by contrast statistics using Student's *t*-tests.

The average hepatic-fat content determined for HC sheep (8.1%; Fig. 4A) was virtually identical to that observed previously (Kalyesubula et al., 2020). Treatment with thiamine, however, had a strong effect on the hepatic-fat content, as it was substantially lower in the THC animals (4.8%, *P*<0.0001; Fig. 4A). Notably, the THC fat content was below the typical 5.5% threshold for fatty liver (Szczepaniak et al., 2005), and statistically similar to the levels in LC sheep (3.9%, *P*=0.164). Hepatocellular steatosis, either macrovesicular or microvesicular, which is correlated with advanced histology of MAFLD (Tandra et al., 2011), was significantly increased in HC compared to both LC and THC animals (Fig. 4B,C). Interestingly, the remarkable reduction in intrahepatic fat caused by thiamine was not associated with a decrease in whole-body insulin resistance (Fig. 3D). Whether the decrease in hepatic fat by thiamine involves a reduction in hepatic insulin resistance remains unknown. This possibility is, however, consistent with the observed increase of hepatic glycogen as a surrogate for insulin sensitivity (Petersen et al., 1998).

Consistent with the previous observation by Kalyesubula et al. (2020), the HC diet increased the hepatic index (liver weight/BW) compared to the LC diet, indicative of disproportional growth of the liver (hepatomegaly). Thiamine, however, had no apparent macroscopic effect on liver growth (Fig. 4D, Table 2) although, microscopically, the size of hepatocytes was increased in THC compared to LC and HC livers (Fig. 4E). Although the mechanisms remain to be investigated, it is plausible that the increase in glycogen content due to thiamine contributed to these observations, since hepatomegaly is associated with increases in either hepatic fat or hepatic glycogen content (Reid, 2001; Sherigar et al., 2018).

### HC diet decreased hepatic expression of genes involved in mitochondrial catabolism

Mammalian regulation of energy expenditure is primarily mediated by sensing of the cellular levels of adenosine monophosphate (AMP) and NAD^+^, respectively, by AMP-activated protein kinase (AMPK) and NAD-dependent protein deacetylase sirtuin-1 (SIRT1). In the setting of low-caloric intake, the resulting increased levels of AMP and NAD^+^ allosterically activate AMPK and SIRT1, respectively. Activation of these evolutionarily-conserved sensors turns on signaling cascades that stimulate catabolic and inhibit anabolic processes. Peroxisome proliferator-activated receptor gamma coactivator-1-alpha (PPARGC1A), a master regulator of mitochondrial biogenesis, is directly targeted by SIRT1 and AMPK to stimulate mitochondrial oxidative processes in response to energy requirements and nutrient availability (Cantó and Auwerx, 2009).

**Fig. 5.**
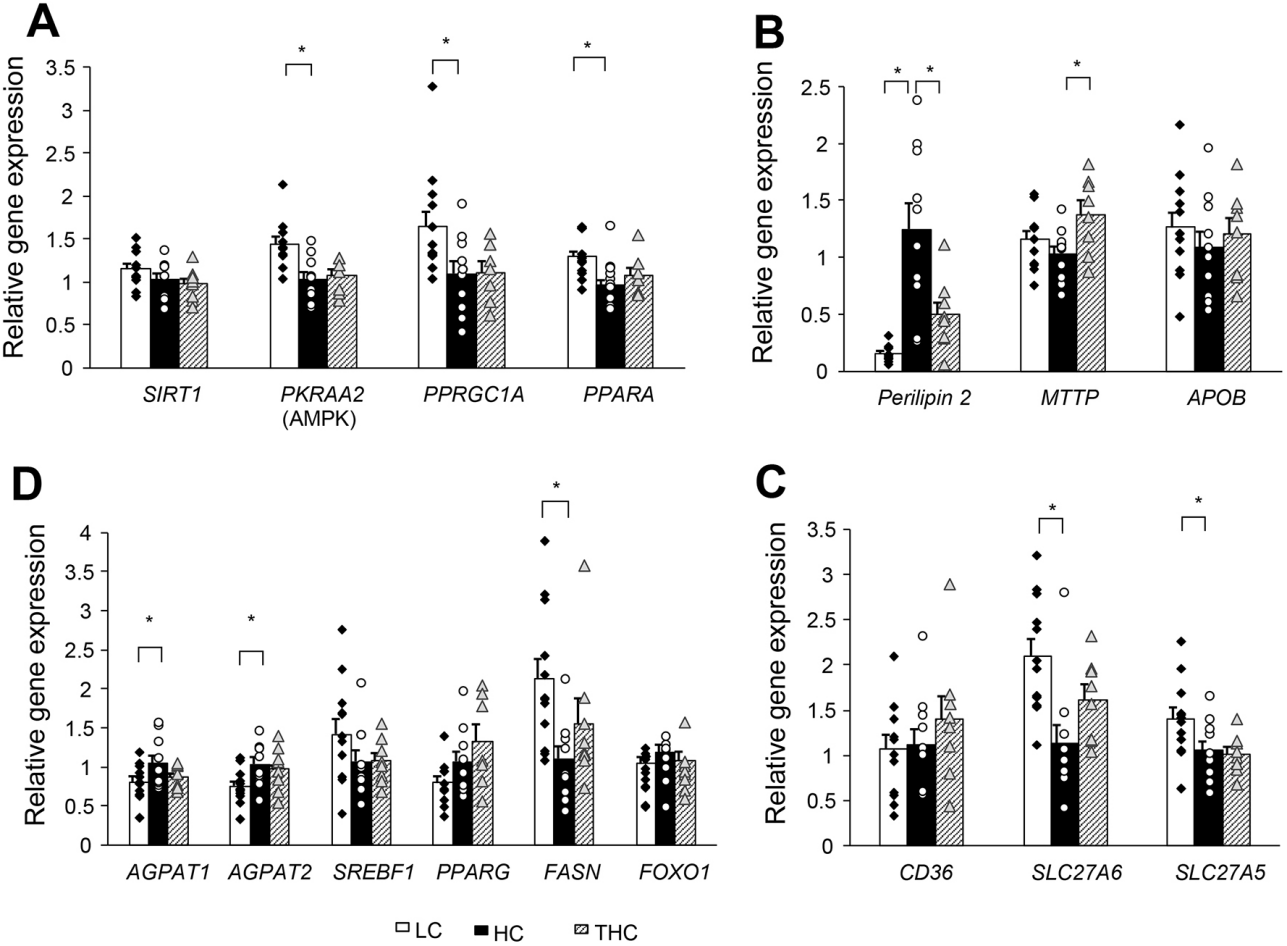
**Expression levels of genes that may affect the hepatic fat content in livers obtained from LC, HC and THC sheep.** (A–D) Expression levels of genes involved in fatty acid oxidation (A), lipid droplet metabolism and VLDL secretion (B), fatty acid uptake (C) and lipogenesis (D). One-way ANOVA detects a treatment effect for the expression of the following genes: *PRKAA2* (*P=*0.002), *PPARGC1A* (*P=*0.03), *PPARA* (*P=*0.009); perilipin 2 (*P<*0.0001), *MTTP* (*P=*0.03), *SLC27A6* (*P=*0.003) and *SLC27A5* (*P=*0.04); *AGPAT2* (*P=*0.04), *PPARG* (*P<*0.05), *FASN* (*P=*0.02). *P<0.05 by contrast statistics using Student's *t*-tests.

In our current study, *SIRT1* expression was similar in the three treatment groups. However, overnourished sheep raised on the HC diet exhibited decreased gene expression of *PRKAA2*, *PPARGC1A* and peroxisome proliferator-activated receptor alpha (*PPARA*) (*P*=0.05; Fig. 5A). Reduced expression of this molecular network, is expected to promote *de novo* lipogenesis and to inhibit fatty acid oxidation (Smith et al., 2016), which is consistent with the increased hepatic steatosis observed in HC animals, both in our present study and in a previous one (Kalyesubula et al., 2020). Thiamine, however, had no effect on the expression levels of these three genes.

### Thiamine-induced alterations in the expression of hepatic genes favored the inhibition of fat storage

Under conditions of energy abundance, as caused by the HC diet, newly derived TG, resulting from hepatic synthesis and liver uptake of circulating lipoproteins, can be either secreted into the bloodstream in very-low-density lipoprotein (VLDL) particles or incorporated into cytosolic lipid droplets (LDs) for storage as intrahepatic fat (Alves-Bezerra and Cohen, 2018). Expansion or reduction of the fat content within LDs is dynamically regulated by LD-associated proteins (Gluchowski et al., 2017), and by the availability of cytosolic TG, which is partly controlled by the lipidation of VLDL particles through microsomal triglyceride transfer protein (MTP) encoded by the *MTTP* gene. Hepatic steatosis has been associated with genetic defects in both MTP and apolipoprotein B-100 (APOB), the hepatic VLDL lipoprotein (Berriot-Varoqueaux et al., 2000; Tanoli et al., 2004). In the current study, thiamine increased the abundance of the *MTTP* transcripts compared with those in the untreated HC group (*P*=0.001; Fig. 5B). No effects were detected on *APOB* transcript levels.

Perilipins are the predominant proteins in hepatocellular LD proteins (Alves-Bezerra and Cohen, 2018). They are associated to the LD surface, stabilizing the structure of LDs and controlling substrate availability for certain LD-associated enzymes (Kimmel and Sztalryd, 2016). Perilipin 2 (PLIN2), which positively correlates with hepatic steatosis (Chang et al., 2006; Fukushima et al., 2005; Motomura et al., 2006; Najt et al., 2016), is one of the best-characterized LD proteins associated with fatty liver disease (Okumura, 2011). We found that, in contrast to animals fed the LC diet, the abundance of perilipin 2 transcripts was significantly increased in those fed the HC diet (*P<*0.0001; Fig. 5B), and that thiamine treatment substantially lowered the transcript levels (*P*=0.002; Fig. 5B). These findings are in accordance with earlier studies, showing that both mRNA and protein levels of PLIN 2 increase with hepatic accumulation of TG (Motomura et al., 2006; Pawella et al., 2014; Straub et al., 2008), whereas inactivation of the perilipin 2 gene lowered hepatic steatosis (Chang et al., 2006; Greenberg et al., 2011).

### Low-calorie diet increased expression of genes involved in liver uptake of fatty acids

A variety of proteins associated with hepatic steatosis have been implicated in the liver uptake of circulating NEFA, including platelet glycoprotein 4 (CD36, also known as fatty acid translocase), caveolin, and fatty acid transport protein (SLC27A, also known as FATP) complexes that possess very-long-chain acyl-CoA synthase activity (Alves-Bezerra and Cohen, 2018). Consistent with the elevated plasma concentrations of NEFA in the LC group (Fig. 3C), abundance of *SLC27A6* (*P*=0.0008) and *SLC27A5* (*P*=0.03) mRNAs was higher in these animals (Fig. 5C). Since PPAR-α positively regulates transcription of NEFA transporters (Pawlak et al., 2015), these data are consistent with the observed increased *PPARA* gene expression of Fig. 5A. Thiamine did not have an effect on the expression of this energy-sensing axis (Fig. 5A), on genes involved in lipogenesis (Fig. 5D) or on NEFA transporters (Fig. 5D).

The low hepatic-fat content in LC lambs (Fig. 4A) suggests, therefore, that influx of NEFA was utilized primarily for energy production, which is consistent with increased expression of genes that promote fatty acid oxidation in LC animals (Fig. 5A).

### Hepatic fat accumulation was associated with altered gene expression of proinflammatory cytokines and antioxidants; some increases in gene expression were reversed by thiamine treatment

**Fig. 6.**
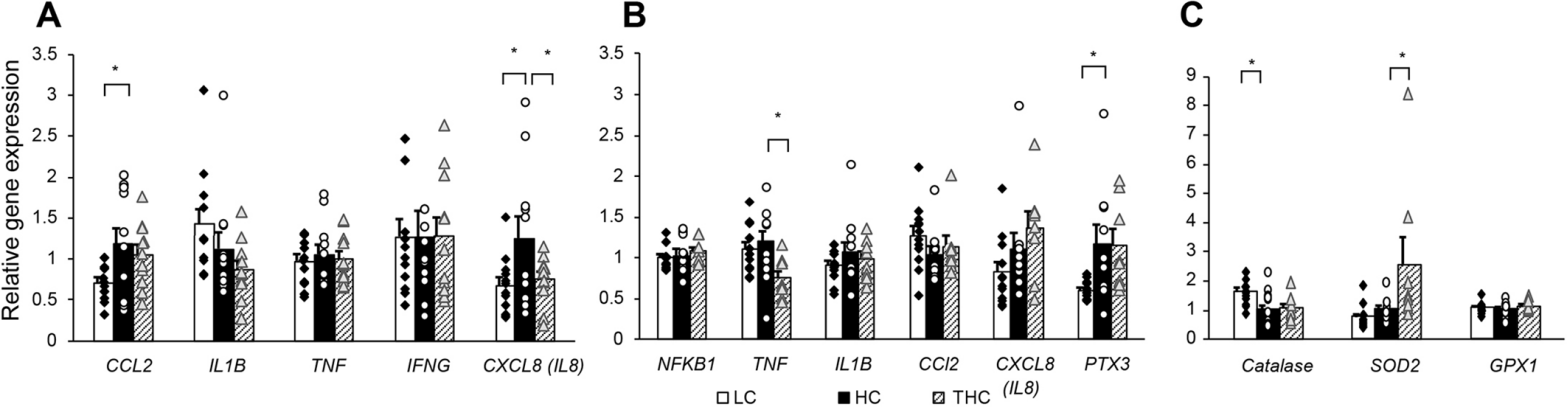
**mRNA quantification of proinflammatory or antioxidant genes in leukocytes or livers from LC-, HC-and THC-treated sheep.** (A–C) Expression levels of proinflammatory factors in leucocytes (A) and livers (B), and of antioxidant enzymes in liver (C) as indicated. One-way ANOVA detected an effect of treatment for leukocytic *CCL2* (*P=*0.04) and *CXCL8* (*P=*0.04), and hepatic *TNF* (*P=*0.01), *PTX3* (*P=*0.02), catalase (*P=*0.008) and *SOD2* (*P=*0.02). *P<0.05 by contrast statistics using Student's *t*-tests.

As a well-documented factor in the pathogenesis of obesity, metabolic syndrome and insulin resistance, inflammation also plays a role in the progression of MAFLD (Ibrahim et al., 2018). In addition to the common histological signs of steatohepatitis, inflammation in MAFLD patients is manifested by increased circulation levels of proinflammatory cytokines and leukocytes that can infiltrate the liver to fuel local and systemic inflammatory processes (Tilg et al., 2020). In our study, HC-fed sheep exhibited increased mRNA levels of C-C motif chemokine 2 and interleukin-8 (*CCL2* and *CXCL8*, respectively) (*P=*0.05; Fig. 6A) in circulating leukocytes as compared to LC-fed animals, but thiamine treatment reversed the dietary effect on *CXCL8* expression (*P=*0.05; Fig. 6A). Leukocytes of HC- or THC-fed animals showed no effect on the expression levels of interleukin-1 beta (*IL1B*), tumor necrosis factor (*TNF*) and interferon gamma (*IFNG*).

In the liver, thiamine lowered the expression of *TNF* (*P=*0.005; Fig. 6B) but did not significantly alter the mRNA levels of *NFKB1*, *IL1B*, *CCL2* and *CXCL8.* The HC diet increased expression of pentraxin 3 (*PTX3*) compared with the LC diet (*P=*0.01; Fig. 6B).

Overnutrition and hepatic steatosis are associated with an increase in reactive oxygen species and oxidative stress, which may decrease levels of endogenous enzymes that act as antioxidants (Spahis et al., 2017; Videla et al., 2004). Consistently, expression of catalase was decreased as a result of the HC diet (*P=*0.005; Fig. 6C) but not affected any further upon treatment with thiamine. Expression levels of superoxide dismutase 2 (*SOD2*) (*P*=0.02; Fig. 6C), however, were increased upon treatment with thiamine. No treatment effects were detected on the expression of glutathione peroxidase 1 (*GPX1*).

## DISCUSSION

Hepatic steatosis is the hallmark of the MAFLD global metabolic epidemic. In this study, we set out to investigate the potential of high-dose thiamine therapy to ameliorate overnutrition-induced hepatic steatosis in a sheep model. The main metabolic phenotypes of this model, i.e. hyperglycemia, hyperinsulinemia, insulin resistance and hepatic steatosis, were virtually identical to those observed in a previous study (Kalyesubula et al., 2020).

We found that thiamine reduced the hepatic-fat content dramatically, decreased blood glucose levels, and increased the hepatic glycogen content. The weekly dose of thiamine employed here, i.e. 900–1500 mg per animal, is likely to be realistic when investigating its effect in other species of similar body weight, i.e. ∼70 kg (Table S2), such as humans. Yet, clearly additional investigations are required to determine the minimal effective dose, duration and frequency of therapy.

Interestingly, the robust effect of thiamine on hepatic steatosis was not accompanied by an effect on whole-body or adipose insulin resistance (Fig. 3D,E), providing evidence that hepatic steatosis may be targeted directly and independently. Whether hepatic insulin resistance is involved in the development of steatosis or modulated by thiamine remains to be investigated. Yet, the increase in hepatic glycogen content upon thiamine treatment (Fig. 3F) is consistent with a reduction in hepatic insulin resistance.

To investigate the involvement of potential metabolic pathways in mediating the observed dietary and thiamine-treatment phenotypes, we performed *ex vivo* studies on postmortem liver extracts. Although these have the capacity to sensitively detect subtle molecular differences between treatments, it is important to realize that postmortem liver extracts reflect a final cumulative picture that highlights lasting and sustained rather than temporary effects.

The change in liver activity of α-KGDH, a key enzyme in setting the TCA-cycle turnover rate, in response to exogenous TPP (Fig. 2B), suggests that animals raised on the HC diet had relatively low levels of hepatic TPP. Consequently, the higher hepatic TPP levels in the thiamine-treated animals may have, potentially, boosted mitochondrial catabolism of pyruvate and acetyl-CoA, by increasing both the catalytic activity of PDH and the oxidative capacity of the TCA cycle. This is consistent with the observed decrease in blood glucose levels and reduced hepatic fat observed in thiamine-treated animals. Interestingly, at gene-expression levels, thiamine treatment might have also increased the glycolytic flux, since abundance of GAPDH mRNA also increased.

In response to cellular energy levels, the AMPK/PPGC-1α/PPAR-α axis orchestrates gene expression to trigger both fatty acid β-oxidation and inhibition of lipogenesis. Whereas the functionality of this energy-sensing axis is mediated by post-translational phosphorylation signals, transcriptional adaptations of the genes involved seems physiologically important and relevant to overnutrition, since decreased expression levels are associated with increased dietary consumption, insulin resistance and metabolic dysfunction (Cantó and Auwerx, 2009; Herzig and Shaw, 2018). Accordingly, strategies to combat insulin resistance, metabolic syndrome and hepatic steatosis have been proposed based on stimulation of this axis (Gariani et al., 2016; Ruderman et al., 2013; Smith et al., 2016).

In our current study, the overnourished animals raised on the HC diet also exhibited decreased expression of AMPK/PPGC-1α/PPAR-α (Fig. 5A), which is consistent with their increased insulin resistance. Moreover, since reduced AMPK signaling is expected to decrease fatty acid oxidation and to increase lipogenesis, these molecular observations are in line with the steatotic phenotype of the HC animals.

Although thiamine robustly decreased the hepatic-fat content (Fig. 4), we found no direct evidence for increased sensitivity to insulin or increased gene expression regarding the AMPK energy-sensing axis (Fig. 5A). This suggests that the mechanism of steatosis reduction or its inhibition by thiamine does not involve insulin sensitization and/or regulation of AMPK signaling on a transcriptional level. This is consistent with the observation that transcription of lipogenic genes was not significantly altered by thiamine (Fig. 5D). The possibility that thiamine is involved in post-translational regulation of AMPK signaling remains to be investigated.

Surprisingly, no effects of thiamine were detected on expression of tested genes involved in lipogenesis and mitochondrial oxidation. However, thiamine-treated animals presented with altered expression of genes involved in LD stability and VLDL lipidation. In particular, transcripts levels of perilipin 2 – which is essential for the structural integrity of LDs and has been shown to be positively correlated with hepatic steatosis (Alves-Bezerra and Cohen, 2018; McIntosh et al., 2012; Motomura et al., 2006; Najt et al., 2016) – were substantially decreased. Notably, this effect of thiamine reversed the strong dietary effect of the HC diet in increasing perilipin 2 transcripts, thus reflecting the observed pattern of steatosis in LC, HC and THC animals. The simultaneous effect of thiamine in increasing mRNA levels of *MTTP*, which is essential for hepatic VLDL lipidation, might further contribute to the depletion of TG from cytosolic LDs and their export to extrahepatic tissues. In line with this, the liver-specific loss of perilipin 2, which alleviates hepatic steatosis, was also associated with an increase in MTP (Najt et al., 2016). The coupling of low levels of PLIN2 with high levels of MTP might serve as a customary module for the efficient depletion of intrahepatic fat.

Progressive liver disease has not been established for the current sheep model, which is more likely to resemble non-alcoholic FL (NAFL). Therefore, it is unlikely that inflammation played a main role in the development of steatosis in this model or in its reversal by thiamine. Nevertheless, since both inflammation and oxidative stress have been extensively documented in obesity and MAFLD (Ibrahim et al., 2018; Tilg et al., 2020), we investigated their association with overnutrition and thiamine intervention, by comparing transcript levels of common proinflammatory cytokines and antioxidants.

Overnourished steatotic sheep displayed increased expression levels of *CCL2* and *CXCL8* in circulating leukocytes, and of *PTX3* in liver. Treatment with thiamine had an effect in the opposite direction, i.e. it reduced the expression of *CXCL8* in leukocytes and *TNF* in liver. Interestingly, treatment of mice with thalidomide, which inhibits production of TNF-α, showed improvements regarding hepatic alterations caused by a high-fat diet (de Fraia Pinto et al., 2010). With regard to oxidative stress, the HC diet decreased the gene expression levels of catalase with thiamine not having any effect; however thiamine did increase the expression of *SOD2*, which, potentially, increases antioxidative capacity.

### Conclusions

In this study, we demonstrated the potential of pharmacological thiamine therapy to address hepatic steatosis resulting from overnutrition. Differential gene expression analyses indicate that reduction of steatosis by thiamine may involve destabilization of LDs and increased VLDL lipidation, rather than insulin sensitization. It will, therefore, be valuable to investigate the clinical benefits of thiamine therapy in the management of hepatic steatosis and of combination therapies using both thiamine and insulin sensitizers for management of FL disorders that involve insulin resistance, such as MAFLD.

## MATERIALS AND METHODS

### Animals and experimental design

All animal studies were approved by the Animal Care Committee of the Volcani Center (Permit 790/18IL), and performed at the Volcani Center experimental sheep farm (Rishon LeZion, Israel). The *in vivo* investigations were started in August and ended in December 2018, corresponding approximately to the end-of-summer through mid-winter in Israel. Animals were maintained in open-shed pens, protected from direct sunlight and rain, with adequate ventilation and daylight. Following weaning at ∼45 days after birth, all male lambs within one crop (a cohort of ∼250 born around June), of the Afec-Assaf breed (Gootwine et al., 2008), were fed a high-calorie (HC) concentrate-based diet (Table S1). Two weeks later, 36 lambs (2.0±0.04 months old, 25.6±0.87 kg body weight) were randomly assigned to three treatment groups (*n*=12 each): (i) HC diet (control for thiamine effects), (ii) HC diet plus thiamine (THC), and (iii) low-calorie (LC) diet (Table S1) as a reference group of lean livers and a control for the dietary effects (see experimental flowchart, Fig. 1). Animals were provided *ad libitum* with their respective group rations, with free access to fresh drinking water. The group feeds and leftovers were weighed weekly. The group means of daily energy intake were 5.16, 5.29 and 3.50 MCal of metabolizable energy for HC, THC and LC animals, respectively (Table S1). These dietary treatments were provided for the entire experimental duration (135 days); pharmacological treatments were given only for the last 85 days.

THC animals were given subcutaneous injections of thiamine hydrochloride (Duchefa Biocemie, Haarlem, The Netherlands) at 300 mg/animal, dissolved in 2 ml of filter-sterilized 0.9% saline solution, administered three times weekly from day 50 onwards and five times weekly from day 93 onwards, to compensate for the increase in the lambs’ body weights. LC and HC lambs were administered with equal volumes of 0.9% saline to control for the potential stress effects of the subcutaneous injection.

### Blood, plasma and liver sampling

Blood was drawn weekly from the jugular vein, and glucose concentrations were measured using a FreeStyle Optium glucometer (Abbot Diabetes Care Ltd., Oxfordshire, UK) (Pichler et al., 2014). Twice monthly, plasma was isolated from 5 ml freshly drawn blood as described (Kalyesubula et al., 2020). On day 130, the animals were fasted for 24 h with free access to water to evaluate blood and plasma fasting parameters. Before fasting, the body condition score (BCS) (Kenyon et al., 2014) was determined, and heart girth, withers height and body length were measured. Fasting plasma samples were stored at −20°C until biochemical analyses. Individual weights were measured weekly.

At day 135, animals were slaughtered at a local abattoir, and their livers immediately harvested and weighed. Samples of ∼10 g from the left lobe of each liver were placed in cryo-tubes, flash-frozen in liquid nitrogen and then stored at −80°C until taken for molecular analyses. Another ∼200 g of the left lobe of each liver was stored in a zip-lock bag at −20°C until used for other analyses. Liver glycogen content was quantified as described (Kalyesubula et al., 2020).

### Fat content analysis

Hepatic fat content analysis was performed using the improved Folch method (Mopuri et al., 2021). Briefly, triplicates of ∼1 g liver tissue were sampled from the frozen left lobe. The exact wet weight of each piece was determined after thawing and dehydrating the excess moisture on a Whatman filter paper for 10 min at 25°C. Each sample was mechanically homogenized in 25 ml chloroform:methanol (2:1) solution, followed by sonication on ice, overnight agitation at 25°C and 10 min centrifugation at 3000 ***g***. For removal of polar and semi-polar lipids, 4 ml of 0.9% NaCl was added to the supernatant, and the mixture was vortexed, then centrifuged at 2500 ***g*** for 10 min. The upper phase was discarded, and the residual interface rinsed twice with 4 ml of 50% methanol. The lower chloroform phase containing the fat (TG and cholesterol esters) was collected and evaporated in a rotary evaporator under vacuum. The residual fatty phase was oven-dried at 45°C for 2.5 h to remove residual moisture. The fat weight was determined, and the hepatic-fat content was computed as the percentage of the wet liver weight.

### Histological analysis

Sample preparation, fixation and staining were performed as described (Kalyesubula et al., 2020). Analyses of Hematoxylin-Eosin (H&E) and periodic acid–Schiff (PAS)-stained sections were examined blindly by a veterinary pathologist. The assessment was performed by counting hepatocytes showing either macrovesicular or microvesicular steatosis at ×200 magnification in three randomly selected fields. Scores of 0, 1, 2 or 3 were assigned to samples presenting <5%, 5–33%, >33–66% or >66% steatotic hepatocytes, respectively. Data are presented on a frequency plot (Fig. 4C), and differences between treatments were explored by univariate χ^2^ analysis.

Assessment of the size of hepatocytes was made by counting their number under a fixed microscopic field at ×200 magnification, which is inversely related to their size. Each sample was observed at three homogeneous zones of liver tissue consisting of hepatocytes and no vessels; the triplicate-average count was used for statistical analysis by ANOVA and contrast statistics using Student's *t*-tests.

### Biochemical analysis of plasma

Plasma TG were measured using the Cobas C 111 analyzer (Roche Diagnostics, Rotkruez, Switzerland). Plasma NEFA concentration was determined using a NEFA kit (Wako Chemicals, GmbH, Neuss, Germany), and plasma insulin was determined using a radioimmunoassay kit (Coat-A-Count insulin; Diagnostic Products, Los Angeles, CA). Plasma thiamine concentrations were analyzed using a Vitamin B1 ELISA kit (Aviva Systems Biology; San Diego, CA). Plasma leptin activity was determined by cell-based bioassay in HEK-293T cells expressing exogenous full-length leptin receptor (*LEPR*) cDNA with firefly luciferase as an intracellular reporter gene, as described (Seroussi et al., 2016).

### Determination of mRNA by quantitative PCR

For isolation of RNA from leukocytes, blood was collected from sheep on day 126 by venipuncture into EDTA-coated tubes and kept on ice. RNA extraction was carried out using the Norgen leukocyte RNA purification kit (Norgen Biotek Corp., Ontario, Canada). DNAase treatment was performed using Promega RQ1 RNAase-Free DNase (Promega, Madison, WI, USA). cDNA was synthesized from 500 ng total RNA using a Revert Aid RT-PCR Kit (Thermo Fisher Scientific, USA).

For isolation of RNA from liver tissue, the Norgen animal tissue RNA purification kit (Norgen Biotek Corp., Ontario, Canada) was used following the manufacturer's instructions. cDNA was synthesized from 1 µg total RNA using a Revert Aid RT-PCR Kit (Thermo Fisher Scientific, USA), using the Applied Biosystems 2720 Thermal Cycler (Thermo Fisher Scientific, USA) following the manufacturer's instructions. RT-qPCR analysis was carried out using 5× HOT FIREPol EvaGreen qPCR Supermix (Solis BioDyne, Tartu, Estonia). The reaction mixture contained: 4 µl of cDNA, 0.3 µl of each primer designed using NCBI Primer Blast (Table S3), 4 µl of 5× HOT FIREPol EvaGreen qPCR Supermix, completed with ultra-pure water (Biological Industries, Kibbutz Beit Ha'emmek, Israel) to a final volume of 20 µl. RT-qPCR was carried out using a Rotor-Gene Q PCR cycler (Qiagen, Hilden, Germany) using the following PCR protocol: 95°C for 12 min, 40 cycles at 95°C for 15 s, 60 cycles at 95°C for 20 s, 72°C for 20 s.

Relative gene expression was computed by using the ΔΔCT method (Livak and Schmittgen, 2001), with the mean of the HC group as the normalizer. For leukocytes, the geometric mean of two reference genes (*GAPDH* and *YWHAZ*; Table S3) was employed; for liver, the geometric mean of three reference genes (*YWHAZ*, *PPIA* and *RPL19*; Table S3) was employed.

### Statistical analysis

Data for continuous dependent variables (glucose, insulin, TG, NEFA concentration and weight; Table 1) were analyzed by repeated measures ANOVA (for variables measured repeatedly over time) with the linear mixed model approach in JMP (Version 14.0.0, SAS Institute Inc., Cary, NC, 2016). The model included Treatment (LC vs HC – for dietary effects) or (THC vs HC – for the thiamine effects) as a between-subject fixed factor, Time (from treatment initiation) as a nominal within-subject fixed factor, Treatment×Time interaction and Individual Animal as a random factor nested within Treatment. For the effect of thiamine, only data obtained when thiamine treatment was initiated (day 50 onwards), were taken into consideration. The distribution of model residuals was visually confirmed for normality. Post-hoc pairwise comparisons between treatments at specific time points were carried out using contrast statistics using Student's *t*-test; significance was corrected for multiple comparisons by using the Holm–Bonferroni method.

Differences between treatments for other response variables that lack the time dimension (as in Table 2), were determined by one-way ANOVA. Selected *a priori* comparisons to investigate the effects of diet (HC vs LC) and of thiamine (HC vs THC) were carried out by using contrast statistics using Student's *t-*tests. Two-tailed *P*-values are reported throughout. Data are presented as means±standard errors (±s.e.), unless otherwise stated. A significance threshold level of α=0.05 was employed.

## Supplementary Material

Supplementary information

## References

[DMM048355C1] Al-Attas, O., Al-Daghri, N., Alokail, M., Abd-Alrahman, S., Vinodson, B. and Sabico, S. (2014). Metabolic benefits of six-month thiamine supplementation in patients with and without diabetes mellitus type 2. *Clin. Med. Insights Endocrinol. Diabetes* 7, 1-6. 10.4137/CMED.S13573 24550684 PMC3921172

[DMM048355C2] Alaei Shahmiri, F., Soares, M. J., Zhao, Y. and Sherriff, J. (2013). High-dose thiamine supplementation improves glucose tolerance in hyperglycemic individuals: a randomized, double-blind cross-over trial. *Eur. J. Nutr.* 52, 1821-1824. 10.1007/s00394-013-0534-6 23715873

[DMM048355C3] Alves-Bezerra, M. and Cohen, D. E. (2018). Triglyceride metabolism in the liver. *Compr. Physiol.* 8, 1-22. 10.1002/cphy.c170012 PMC6376873 29357123

[DMM048355C4] Arvidsson, A., Collin, T., Kirk, D., Kokaia, Z. and Lindvall, O. (2003). Neuronal replacement from endogenous precursors in the adult brain after stroke. *Nat. Med.* 9, 548-553. 10.1038/nm747 12161747

[DMM048355C5] Asselah, T., Rubbia-Brandt, L., Marcellin, P. and Negro, F. (2006). Steatosis in chronic hepatitis C: Why does it really matter? *Gut* 55, 123-130. 10.1136/gut.2005.069757 16344578 PMC1856395

[DMM048355C6] Babaei-Jadidi, R., Karachalias, N., Kupich, C., Ahmed, N. and Thornalley, P. J. (2004). High-dose thiamine therapy counters dyslipidaemia in streptozotocin-induced diabetic rats. *Diabetologia* 47, 2235-2246. 10.1007/s00125-004-1582-5 15662560

[DMM048355C7] Berriot-Varoqueaux, N., Aggerbeck, L. P., Samson-Bouma, M.-E. and Wetterau, J. R. (2000). The role of the microsomal triglygeride transfer protein in abetalipoproteinemia. *Annu. Rev. Nutr.* 20, 663-697. 10.1146/annurev.nutr.20.1.663 10940349

[DMM048355C8] Bocobza, S. E., Malitsky, S., Araújo, W. L., Nunes-Nesi, A., Meir, S., Shapira, M., Fernie, A. R. and Aharoni, A. (2013). Orchestration of thiamin biosynthesis and central metabolism by combined action of the thiamin pyrophosphate riboswitch and the circadian clock in arabidopsis. *Plant Cell* 25, 288-307. 10.1105/tpc.112.106385 23341335 PMC3584542

[DMM048355C9] Cantó, C. and Auwerx, J. (2009). PGC-1α, SIRT1 and AMPK, an energy sensing network that controls energy expenditure. *Curr. Opin. Lipidol.* 20, 98-105. 10.1097/MOL.0b013e328328d0a4 19276888 PMC3627054

[DMM048355C10] Chang, B. H.-J., Li, L., Paul, A., Taniguchi, S., Nannegari, V., Heird, W. C. and Chan, L. (2006). Protection against fatty liver but normal adipogenesis in mice lacking adipose differentiation-related protein. *Mol. Cell. Biol.* 26, 1063-1076. 10.1128/MCB.26.3.1063-1076.2006 16428458 PMC1347045

[DMM048355C11] de Fraia Pinto, L., Compri, C. M., Fornari, J. V., Bartchewsky, W., Cintra, D. E., Trevisan, M., de Olivera Carvalho, P., Ribeiro, M. L., Velloso, L. A., Saad, M. J. et al. (2010). The immunosuppressant drug, thalidomide, improves hepatic alterations induced by a high-fat diet in mice. *Liver Int.* 30, 603-610. 10.1111/j.1478-3231.2009.02200.x 20088867

[DMM048355C12] Elmadfa, I., Majchrzak, D., Rust, P. and Genser, D. (2001). The thiamine status of adult humans depends on carbohydrate intake. *Int. J. Vitam. Nutr. Res.* 71, 217-221. 10.1024/0300-9831.71.4.217 11582856

[DMM048355C13] Eshak, E. S. and Arafa, A. E. (2018). Thiamine deficiency and cardiovascular disorders. *Nutr. Metab. Cardiovasc. Dis.* 28, 965-972. 10.1016/j.numecd.2018.06.013 30143411

[DMM048355C14] Eslam, M., Newsome, P. N., Sarin, S. K., Anstee, Q. M., Targher, G., Romero-Gomez, M., Zelber-Sagi, S., Wai-Sun Wong, V., Dufour, J. F., Schattenberg, J. M. et al. (2020a). A new definition for metabolic dysfunction-associated fatty liver disease: An international expert consensus statement. *J. Hepatol.* 73, 202-209. 10.1016/j.jhep.2020.03.039 32278004

[DMM048355C15] Eslam, M., Sanyal, A. J., George, J., Sanyal, A., Neuschwander-Tetri, B., Tiribelli, C., Kleiner, D. E., Brunt, E., Bugianesi, E., Yki-Järvinen, H. et al. (2020b). MAFLD: a consensus-driven proposed nomenclature for metabolic associated fatty liver disease. *Gastroenterology* 158, 1999-2014.e1. 10.1053/j.gastro.2019.11.312 32044314

[DMM048355C16] Friedman, S. L., Neuschwander-Tetri, B. A., Rinella, M. and Sanyal, A. J. (2018). Mechanisms of NAFLD development and therapeutic strategies. *Nat. Med.* 24, 908-922. 10.1038/s41591-018-0104-9 29967350 PMC6553468

[DMM048355C17] Fukushima, M., Enjoji, M., Kohjima, M., Sugimoto, R., Ohta, S., Kotoh, K., Kuniyoshi, M., Kobayashi, K., Imamura, M., Inoguchi, T. et al. (2005). Adipose differentiation related protein induces lipid accumulation and lipid droplet formation in hepatic stellate cells. *Vitr. Cell. Dev. Biol. Anim.* 41, 321-324. 10.1007/s11626-005-0002-6 16448220

[DMM048355C18] Gariani, K., Menzies, K. J., Ryu, D., Wegner, C. J., Wang, X., Ropelle, E. R., Moullan, N., Zhang, H., Perino, A., Lemos, V. et al. (2016). Eliciting the mitochondrial unfolded protein response by nicotinamide adenine dinucleotide repletion reverses fatty liver disease in mice. *Hepatology* 63, 1190-1204. 10.1002/hep.28245 26404765 PMC4805450

[DMM048355C19] Gluchowski, N. L., Becuwe, M., Walther, T. C. and Farese, R. V. (2017). Lipid droplets and liver disease: from basic biology to clinical implications. *Nat. Rev. Gastroenterol. Hepatol.* 14, 343-355. 10.1038/nrgastro.2017.32 28428634 PMC6319657

[DMM048355C20] Goldberg, D., Ditah, I. C., Saeian, K., Lalehzari, M., Aronsohn, A., Gorospe, E. C. and Charlton, M. (2017). Changes in the prevalence of Hepatitis C virus infection, nonalcoholic steatohepatitis, and alcoholic liver disease among patients with cirrhosis or liver failure on the waitlist for liver transplantation. *Gastroenterology* 152, 1090-1099.e1. 10.1053/j.gastro.2017.01.003 28088461 PMC5367965

[DMM048355C21] Gootwine, E., Reicher, S. and Rozov, A. (2008). Prolificacy and lamb survival at birth in Awassi and Assaf sheep carrying the FecB (Booroola) mutation. *Anim. Reprod. Sci.* 108, 402-411. 10.1016/j.anireprosci.2007.09.009 17997056

[DMM048355C22] Gorlova, A., Pavlov, D., Anthony, D. C., Ponomarev, E. D., Sambon, M., Proshin, A., Shafarevich, I., Babaevskaya, D., Lesсh, K. P., Bettendorff, L. et al. (2019). Thiamine and benfotiamine counteract ultrasound-induced aggression, normalize AMPA receptor expression and plasticity markers, and reduce oxidative stress in mice. *Neuropharmacology* 156, 107543. 10.1016/j.neuropharm.2019.02.025 30817932

[DMM048355C23] Greenberg, A. S., Coleman, R. A., Kraemer, F. B., McManaman, J. L., Obin, M. S., Puri, V., Yan, Q.-W., Miyoshi, H. and Mashek, D. G. (2011). The role of lipid droplets in metabolic disease in rodents and humans. *J. Clin. Invest.* 121, 2102-2110. 10.1172/JCI46069 21633178 PMC3104768

[DMM048355C24] Hammes, H.-P., Du, X., Edelstein, D., Taguchi, T., Matsumura, T., Ju, Q., Lin, J., Bierhaus, A., Nawroth, P., Hannak, D. et al. (2003). Benfotiamine blocks three major pathways of hyperglycemic damage and prevents experimental diabetic retinopathy. *Nat. Med.* 9, 294-299. 10.1038/nm834 12592403

[DMM048355C25] Herzig, S. and Shaw, R. J. (2018). AMPK: Guardian of metabolism and mitochondrial homeostasis. *Nat. Rev. Mol. Cell Biol.* 19, 121-135. 10.1038/nrm.2017.95 28974774 PMC5780224

[DMM048355C26] Huang, H.-M., Zhang, H., Xu, H. and Gibson, G. E. (2003). Inhibition of the α-ketoglutarate dehydrogenase complex alters mitochondrial function and cellular calcium regulation. *Biochim. Biophys. Acta Mol. Basis Dis.* 1637, 119-126. 10.1016/S0925-4439(02)00222-3 12527416

[DMM048355C27] Ibrahim, S. H., Hirsova, P. and Gores, G. J. (2018). Non-alcoholic steatohepatitis pathogenesis: sublethal hepatocyte injury as a driver of liver inflammation. *Gut* 67, 963-972. 10.1136/gutjnl-2017-315691 29367207 PMC5889737

[DMM048355C28] Kalyesubula, M., Mopuri, R., Rosov, A., Alon, T., Edery, N., Moallem, U. and Dvir, H. (2020). Hyperglycemia-stimulating diet induces liver steatosis in sheep. *Sci. Rep.* 10, 12189. 10.1038/s41598-020-68909-z 32699301 PMC7376193

[DMM048355C29] Karachalias, N., Babaei-Jadidi, R., Rabbani, N. and Thornalley, P. J. (2010). Increased protein damage in renal glomeruli, retina, nerve, plasma and urine and its prevention by thiamine and benfotiamine therapy in a rat model of diabetes. *Diabetologia* 53, 1506-1516. 10.1007/s00125-010-1722-z 20369223

[DMM048355C30] Kawano, Y. and Cohen, D. E. (2013). Mechanisms of hepatic triglyceride accumulation in non-alcoholic fatty liver disease. *J. Gastroenterol.* 48, 434-441. 10.1007/s00535-013-0758-5 23397118 PMC3633701

[DMM048355C31] Kenyon, P. R., Maloney, S. K. and Blache, D. (2014). Review of sheep body condition score in relation to production characteristics. *New Zeal. J. Agric. Res.* 57, 38-64. 10.1080/00288233.2013.857698

[DMM048355C32] Kimmel, A. R. and Sztalryd, C. (2016). the perilipins: major cytosolic lipid droplet–associated proteins and their roles in cellular lipid storage, mobilization, and systemic homeostasis. *Annu. Rev. Nutr.* 36, 471-509. 10.1146/annurev-nutr-071813-105410 27431369

[DMM048355C33] Livak, K. J. and Schmittgen, T. D. (2001). Analysis of relative gene expression data using real-time quantitative PCR and the 2−ΔΔCT method. *Methods* 25, 402-408. 10.1006/meth.2001.1262 11846609

[DMM048355C34] Lonsdale, D. (2006). A review of the biochemistry, metabolism and clinical benefits of thiamin(e) and its *derivatives*. *Evid. Based Complement. Altern. Med.* 3, 49-59. 10.1093/ecam/nek009 PMC1375232 16550223

[DMM048355C35] Louvet, A. and Mathurin, P. (2015). Alcoholic liver disease: mechanisms of injury and targeted treatment. *Nat. Rev. Gastroenterol. Hepatol.* 12, 231-242. 10.1038/nrgastro.2015.35 25782093

[DMM048355C36] Maguire, D., Talwar, D., Shiels, P. G. and McMillan, D. (2018). The role of thiamine dependent enzymes in obesity and obesity related chronic disease states: a systematic review. *Clin. Nutr. ESPEN* 25, 8-17. 10.1016/j.clnesp.2018.02.007 29779823

[DMM048355C37] Manzetti, S., Zhang, J. and Van Der Spoel, D. (2014). Thiamin function, metabolism, uptake, and transport. *Biochemistry* 53, 821-835. 10.1021/bi401618y 24460461

[DMM048355C38] Mastrogiacomo, F., Bergeron, C. and Kish, S. J. (1993). Brain α-ketoglutarate dehydrogenase complex activity in Alzheimer's disease. *J. Neurochem.* 61, 2007-2014. 10.1111/j.1471-4159.1993.tb07436.x 8245957

[DMM048355C39] McIntosh, A. L., Senthivinayagam, S., Moon, K. C., Gupta, S., Lwande, J. S., Murphy, C. C., Storey, S. M. and Atshaves, B. P. (2012). Direct interaction of Plin2 with lipids on the surface of lipid droplets: a live cell FRET analysis. *Am. J. Physiol. Cell Physiol.* 303, C728-C742. 10.1152/ajpcell.00448.2011 22744009 PMC3469596

[DMM048355C40] Mittal, S., El-Serag, H. B., Sada, Y. H., Kanwal, F., Duan, Z., Temple, S., May, S. B., Kramer, J. R., Richardson, P. A. and Davila, J. A. (2016). Hepatocellular carcinoma in the absence of cirrhosis in united states veterans is associated with nonalcoholic fatty liver disease. *Clin. Gastroenterol. Hepatol.* 14, 124-131.e1. 10.1016/j.cgh.2015.07.019 26196445 PMC4690789

[DMM048355C41] Mopuri, R., Kalyesubula, M., Rosov, A., Edery, N., Moallem, U. and Dvir, H. (2021). Improved Folch method for liver-fat quantification. *Front. Vet. Sci*. 7, 3-7. 10.3389/fvets.2020.594853 PMC7835396 33511163

[DMM048355C42] Motomura, W., Inoue, M., Ohtake, T., Takahashi, N., Nagamine, M., Tanno, S., Kohgo, Y. and Okumura, T. (2006). Up-regulation of ADRP in fatty liver in human and liver steatosis in mice fed with high fat diet. *Biochem. Biophys. Res. Commun.* 340, 1111-1118. 10.1016/j.bbrc.2005.12.121 16403437

[DMM048355C43] Najt, C. P., Senthivinayagam, S., Aljazi, M. B., Fader, K. A., Olenic, S. D., Brock, J. R. L., Lydic, T. A., Jones, A. D. and Atshaves, B. P. (2016). Liver-specific loss of Perilipin 2 alleviates diet-induced hepatic steatosis, inflammation, and fibrosis. *Am. J. Physiol. Gastrointest. Liver Physiol.* 310, G726-G738. 10.1152/ajpgi.00436.2015 26968211 PMC4867327

[DMM048355C44] Okumura, T. (2011). Role of lipid droplet proteins in liver steatosis. *J. Physiol. Biochem.* 67, 629-636. 10.1007/s13105-011-0110-6 21847662

[DMM048355C45] Pawella, L. M., Hashani, M., Eiteneuer, E., Renner, M., Bartenschlager, R., Schirmacher, P. and Straub, B. K. (2014). Perilipin discerns chronic from acute hepatocellular steatosis. *J. Hepatol.* 60, 633-642. 10.1016/j.jhep.2013.11.007 24269473

[DMM048355C46] Pawlak, M., Lefebvre, P. and Staels, B. (2015). Molecular mechanism of PPARα action and its impact on lipid metabolism, inflammation and fibrosis in non-alcoholic fatty liver disease. *J. Hepatol.* 62, 720-733. 10.1016/j.jhep.2014.10.039 25450203

[DMM048355C47] Petersen, K. F., Laurent, D., Rothman, D. L., Cline, G. W. and Shulman, G. I. (1998). Mechanism by which glucose and insulin inhibit net hepatic glycogenolysis in humans. *J. Clin. Invest.* 101, 1203-1209. 10.1172/JCI579 9502760 PMC508673

[DMM048355C48] Pichler, M., Damberger, A., Schwendenwein, I., Gasteiner, J., Drillich, M. and Iwersen, M. (2014). Thresholds of whole-blood β-hydroxybutyrate and glucose concentrations measured with an electronic hand-held device to identify ovine hyperketonemia. *J. Dairy Sci.* 97, 1388-1399. 10.3168/jds.2013-7169 24440266

[DMM048355C49] Rabbani, N., Alam, S. S., Riaz, S., Larkin, J. R., Akhtar, M. W., Shafi, T. and Thornalley, P. J. (2009). High-dose thiamine therapy for patients with type 2 diabetes and microalbuminuria: a randomised, double-blind placebo-controlled pilot study. *Diabetologia* 52, 208-212. 10.1007/s00125-008-1224-4 19057893

[DMM048355C50] Reid, A. E. (2001). Nonalcoholic steatohepatitis. *Gastroenterology* 121, 710-723. 10.1053/gast.2001.27126 11522755

[DMM048355C51] Roach, P. J., Depaoli-Roach, A. A., Hurley, T. D. and Tagliabracci, V. S. (2012). Glycogen and its metabolism: some new developments and old themes. *Biochem. J.* 441, 763-787. 10.1042/BJ20111416 22248338 PMC4945249

[DMM048355C52] Ruderman, N. B., Carling, D., Prentki, M. and Cacicedo, J. M. (2013). AMPK, insulin resistance, and the metabolic syndrome. *J. Clin. Invest.* 123, 2764-2772. 10.1172/JCI67227 23863634 PMC3696539

[DMM048355C53] Samuel, V. T. and Shulman, G. I. (2016). The pathogenesis of insulin resistance: integrating signaling pathways and substrate flux. *J. Clin. Invest.* 126, 12-22. 10.1172/JCI77812 26727229 PMC4701542

[DMM048355C54] Santhekadur, P. K., Kumar, D. P. and Sanyal, A. J. (2018). Preclinical models of non-alcoholic fatty liver disease. *J. Hepatol.* 68, 230-237. 10.1016/j.jhep.2017.10.031 29128391 PMC5775040

[DMM048355C55] Seroussi, E., Cinnamon, Y., Yosefi, S., Genin, O., Smith, J. G., Rafati, N., Bornelöv, S., Andersson, L. and Friedman-Einat, M. (2016). Identification of the long-sought leptin in chicken and duck: expression pattern of the highly GC-rich avian leptin fits an autocrine/paracrine rather than endocrine function. *Endocrinology* 157, 737-751. 10.1210/en.2015-1634 26587783

[DMM048355C56] Sherigar, J. M., de Castro, J., Yin, Y. M., Guss, D. and Mohanty, S. R. (2018). Glycogenic hepatopathy: a narrative review. *World J. Hepatol.* 10, 172-185. 10.4254/wjh.v10.i2.172 29527255 PMC5838438

[DMM048355C57] Smith, B. K., Marcinko, K., Desjardins, E. M., Lally, J. S., Ford, R. J. and Steinberg, G. R. (2016). Treatment of nonalcoholic fatty liver disease: role of AMPK. *Am. J. Physiol. Metab.* 311, E730-E740. 10.1152/ajpendo.00225.2016 27577854

[DMM048355C58] Spahis, S., Delvin, E., Borys, J.-M. and Levy, E. (2017). Oxidative stress as a critical factor in nonalcoholic fatty liver disease pathogenesis. *Antioxid. Redox Signal.* 26, 519-541. 10.1089/ars.2016.6776 27452109

[DMM048355C59] Straub, B. K., Stoeffel, P., Heid, H., Zimbelmann, R. and Schirmacher, P. (2008). Differential pattern of lipid droplet-associated proteins and de novo perilipin expression in hepatocyte steatogenesis. *Hepatology* 47, 1936-1946. 10.1002/hep.22268 18393390

[DMM048355C60] Szczepaniak, L. S., Nurenberg, P., Leonard, D., Browning, J. D., Reingold, J. S., Grundy, S., Hobbs, H. H. and Dobbins, R. L. (2005). Magnetic resonance spectroscopy to measure hepatic triglyceride content: Prevalence of hepatic steatosis in the general population. *Am. J. Physiol. Endocrinol. Metab.* 288, E462-E468. 10.1152/ajpendo.00064.2004 15339742

[DMM048355C61] Tandra, S., Yeh, M. M., Brunt, E. M., Vuppalanchi, R., Cummings, O. W., Ünalp-Arida, A., Wilson, L. A. and Chalasani, N. (2011). Presence and significance of microvesicular steatosis in nonalcoholic fatty liver disease. *J. Hepatol.* 55, 654-659. 10.1016/j.jhep.2010.11.021 21172393 PMC3139780

[DMM048355C62] Tanoli, T., Yue, P., Yablonskiy, D. and Schonfeld, G. (2004). Fatty liver in familial hypobetalipoproteinemia. *J. Lipid Res.* 45, 941-947. 10.1194/jlr.M300508-JLR200 14967820

[DMM048355C63] Thornalley, P. J. and Ali, H. A. (2007). High prevalence of low plasma thiamine concentration in diabetes linked to a marker of vascular disease. 50, 2164-2170. 10.1007/s00125-007-0771-4 PMC1998885 17676306

[DMM048355C64] Tilg, H., Adolph, T. E. and Moschen, A. R. (2020). Multiple parallel hits hypothesis in NAFLD – revisited after a decade. *Hepatology*, 73, 833-842. 10.1002/hep.31518 PMC7898624 32780879

[DMM048355C65] Tunc-Ozdemir, M., Miller, G., Song, L., Kim, J., Sodek, A., Koussevitzky, S., Misra, A. N., Mittler, R. and Shintani, D. (2009). Thiamin confers enhanced tolerance to oxidative stress in Arabidopsis. *Plant Physiol.* 151, 421-432. 10.1104/pp.109.140046 19641031 PMC2735988

[DMM048355C66] Videla, L. A., Rodrigo, R., Orellana, M., Fernandez, V., Tapia, G., Quiñones, L., Varela, N., Contreras, J., Lazarte, R., Csendes, A. et al. (2004). Oxidative stress-related parameters in the liver of non-alcoholic fatty liver disease patients. *Clin. Sci. (Lond).* 106, 261-268. 10.1042/CS20030285 14556645

[DMM048355C67] Voskoboyev, A. I. and Ostrovsky, Y. M. (1982). Thiamin pyrophosphokinase: structure, properties and role in thiamin metabolism. *Ann. NY Acad. Sci.* 378, 161-176. 10.1111/j.1749-6632.1982.tb31195.x 6282163

[DMM048355C68] Wong, R. J., Aguilar, M., Cheung, R., Perumpail, R. B., Harrison, S. A., Younossi, Z. M. and Ahmed, A. (2015). Nonalcoholic steatohepatitis is the second leading etiology of liver disease among adults awaiting liver transplantation in the United States. *Gastroenterology* 148, 547-555. 10.1053/j.gastro.2014.11.039 25461851

[DMM048355C69] Younossi, Z., Anstee, Q. M., Marietti, M., Hardy, T., Henry, L., Eslam, M., George, J. and Bugianesi, E. (2018). Global burden of NAFLD and NASH: Trends, predictions, risk factors and prevention. *Nat. Rev. Gastroenterol. Hepatol.* 15, 11-20. 10.1038/nrgastro.2017.109 28930295

