# Supplementary material for: High-dose vitamin B1 therapy prevents the development of experimental fatty liver driven by overnutrition
Source: Dis Model Mech. 2021 Mar 18;14(3):dmm048355. doi: 10.1242/dmm.048355 (PMC7988776; doi:10.1242/dmm.048355)
Supplement: Supplementary information [file dmm-14-048355-s1.pdf]

Supplementary information

Table S1. Composition of feeds and intake for the low-calorie (LC), high-calorie (HC), and thiamine-treated HC (THC) sheep, expressed as dry matter.

Composition	Oat Hay	Concentrate	Ground Corn	Soybean meal	
Moisture (%)	11.0	14.9	16.3	13.6	
Crude protein (%)	10.0	18.4	9.4	53.0	
Crude Fat (%)	3	3.5	4.1	1.6	
NDF (%)	56.0	22.6	9.2	8.0	
ADF (%)	31.6	9.9	2.6	5.9	
ME, MCal/kg	2.2	3.2	3.9	2.8	
					**Total Intake**
**LC** average intake (Kg) ± SD.	0.96±0.35	-	0.28±0.08	0.27±0.08	1.52±0.48
LC average intake (ME, MCal/kg)	1.87±0.67	-	0.95±0.27	0.68±0.19	3.50±1.07
**HC** average intake (Kg) ± SD.	0.09±0.02	1.79±0.28	-	-	1.88±0.28
HC average intake (ME, MCal/Kg)	0.17±0.03	5.00±0.79	-	-	5.16±0.78
**THC** average intake (Kg) ± SD.	0.09±0.01	1.83±0.29	-		1.93±0.28
THC average intake (ME, MCal/Kg)	0.18±0.03	5.11±0.80	-	-	5.29±0.79

Table S2. Physical body and blood plasma parameters for the low calorie (LC), high calorie (HC), and thiamine treated-high calorie (THC) sheep.

	LC	HC	THC	SEM	*P*-values		
					ANOVA	Dietary effects[1]	Thiamine effects[2]
Initial BW (kg)	25.8	25.2	25.8	0.9	0.94	0.76	0.77
Final BW (kg)	47.5	71.3	70.4	2.1	<.0001	<.0001	0.70
Body length (m)	0.85	0.98	0.93	0.01	<.0001	<.0001	0.02
Wither's height (m)	0.69	0.74	0.74	0.006	<.0001	<.0001	0.7
Heart girth (cm)	89.0	103.3	102.3	1.4	<.0001	<.0001	0.61
Fasting Body weight, kg	44.5	72.5	68.5	2.3	<.0001	<.0001	0.11
Fasting Weight loss, kg	-1.08	-0.18	-0.78	0.14	0.03	0.009	0.08
Fasting Plasma TG, mg/dL	20.1	20.5	16.8	1.0	0.28	0.85	0.15

BW: body weight, BL: body length, SEM: Standard error of mean.

[1]Dietary effects were obtained from contrast *t*-test between HC and LC animals.

[2]Thiamine effects were obtained from contrast *t*-test between THC and HC animals.

Table S3. Primer sequences used for real-time quantitative PCR

Gene	GenBank Accession	Primer	Length (bp)
**Leukocytes**			
GAPDH	NM_001190390.1	Forward: AAGTTCCACGGCACAGTCAA<br>Reverse: ATGTTGGCAGGATCTCGCTC	92
YWHAZ	XM_027972757.1	Forward: AGACGGAAGGTGCTGAGAAA<br>Reverse: CGTTGGGGATCAAGAACTTT	123
TNF	NM_001024860.1	Forward: CACGTTGTAGCCAACATCAGC<br>Reverse: GAGGTAAAGCCCGTCAGTGG	129
CCL2	XM_004012471.2	Forward: TCGCTCAGCCAGATGCAATTA<br>Reverse: GACACTTGCTGGTGGTGACT	112
CXCL8 (IL8)	NM_001009401.2	Forward: AAGCTGGCTGTTGCTCTCTTG<br>Reverse: GTGGAAAGGTGTGGAATGTGT	127
IFNG	X52640	Forward: GGAGGACTTCAAAAGGCTGA<br>Reverse: GGTTAGATTTTGGCGACAGG	110
IL1B	NM_001009465.2	Forward: GTGCTGGATAGCCCATGTGT<br>Reverse: CAGAACACCACTTCTCGGCT	74
**Liver**			
AGPAT1	NM_001009746.1	Forward: TTGCGTCTGATGCTACTCCA<br>Reverse: CTCCATCATCCCAAGCAGGTC	141
AGPAT2	XM_015094112.2	Forward: CGGTGGAGAACATGAGCATC<br>Reverse: CGGTGGAGAACATGAGCATC	138
SREBF1	XM_027974781.1	Forward: GAGCTTCGTGGTTTCCAGA<br>Reverse: TGTAGGAATACCCTCCGCAT	86
PPARG	NM_001100921.1	Forward: GACGACAGACAAATCACCGT<br>Reverse: GGGGCTGATGTGCTTGAACT	88
FOXO1	XM_027973596.1	Forward: ATCGCAGTTTTCCAAGTGGC<br>Reverse: ACTAGCGTTCGAGCTGGTTC	100
FASN	AF479289.1	Forward: CCCAGCTCAACGAAACCA<br>Reverse: GACGAGGTCAACACCCTTCC	110
SIRT1	XM_015104377.2	Forward: TTGGGTACCGAGATGACCTT<br>Reverse: GCATGCGAGGCTCTATCATC	94
PPARA	XM_012145989.2	Forward: GTCCCATAACGCGATTCGT<br>Reverse: GATCTGCGGTTTCGGAATCT	110
PRKAA2	NM_001112816.1	Forward: GTCATCTCAGGCAGGCTGTA<br>Reverse: AGGGTGCCACAAAGAAGAGC	87
PPARGC1A	XM_004009738.4	Forward: AGTGACATCGAGTGTGCTGC<br>Reverse: CTGTCTGTATCCAAGTCGTTC	109
Perilipin 2	NM_001104932.1	Forward: ACAGCTGAGTGTGGTGACAA<br>Reverse: CTGATGTAAGCCGAGGACAC	82
Catalase	XM_004016396.4	Forward: GCAACTACCCCTCCTGGACT<br>Reverse: CACTGTGAGGCCAAACCTTAG	105
SOD2	NM_001280703.1	Forward: CAATAAGGAGCAGGGACGCT<br>Reverse: TAAGCATGCTCCCACACGTC	111
GPX1	XM_004018462.4	Forward: CACCCAGATGAATGACCTGC<br>Reverse: CGTTCTTGGCGTTTTCCTGA	102
Glycogen synthase 2	XM_004006794.4	Forward: CAACTCAGCGGCAGTCTTTG<br>Reverse: TGACTCTGTCTGTGCGGCTA	120

Disease Models & Mechanisms • Supplementary information

PYGL	NM_001024861.1	Forward: CAGCCAGCTGTACATGAATCC<br>Reverse: CCCGGGCGTACTCCTTAATC	107
GSK3B	XM_012178649.3	Forward: CGAGACACACCTGCACTCTT<br>Reverse: GCATTAGCATCTGAGGCTGC	146
NFKB1	XM_004009667.3	Forward: GCAGATGGCCCATACCTTCAA<br>Reverse: GTTGCAGATTTTGACCTGAGGG	150
TNF	NM_001024860.1	Forward: CACGTTGTAGCCAACATCAGC<br>Reverse: GAGGTAAAGCCCGTCAGTGG	129
IL1B	NM_001009465.2	Forward: GTGCTGGATAGCCCATGTGT<br>Reverse: CAGAACACCACTTCTCGGCT	74
CCL2	XM_004012471.2	Forward: TCGCTCAGCCAGATGCAATTA<br>Reverse: GACACTTGCTGGTGGTGACT	112
CXCL8 (IL8)	NM_001009401.2	Forward: AAGCTGGCTGTTGCTCTCTTG<br>Reverse: GTGGAAAGGTGTGGAATGTGT	127
PTX3	AM492193.1	Forward: CCTGCATTTGGGTCAAAGCC<br>Reverse: CTCCACCCACCACAAGCATT	129
MTTP	XM_027970731.1	Forward: GAGCTGAAAACCACGGAAGC<br>Reverse: TCTGAGAGAGAAGGGCATCCT	133
APOB	XM_027966544.1	Forward: TGTATGGCCTCGATCCTGAG<br>Reverse: GGTCATACTTGGACATGGCA	88
CD36	XM_027968558.1	Forward: ATGGTACAGATGCAGCCTCA<br>Reverse: ATGGTACAGATGCAGCCTCA	105
SLC27A5	XM_027978950.1	Forward: TTCGTATCCAGGACGCCTT<br>Reverse: GAACAGAGGATCAGCGACGA	104
SLC27A6	XM_004008652.4	Forward: TTGGAGACACTTTCAGGTGG<br>Reverse: CTGGCACAACCACACCATAG	114
Insulin Receptor	XM_004008549.4	Forward: ACGCCACTAATCCTTCCGTC<br>Reverse: AGGGAGGCTTCCACTTCAGA	84
IGF1R	XM_027957015.1	Forward: GACGTGGCAATAACATCGCT<br>Reverse: GAGTGGCGGATCTTCACGTA	112
GAPDH	NM_001190390.1	Forward: TCGGAGTGAACGGATTTGGC<br>Reverse: ACGATGTCCACTTTGCCAGT	76
PPIA	NM_001308578.1	Forward: TTATGTGCCAGGGTGGTGAC<br>Reverse: GCCAGGACCTGTATGCTTCA	110
YWHAZ	XM_027972757.1	Forward: ACTGGGTCTGGCCCTTAAC<br>Reverse: CGATGTCCACAATGTCAAGT	190
RPL19	AY158223.1	Forward: AGCCTGTGACTGTCCATTCC<br>Reverse: ACCTATACCCATATGCCTGCC	80

Abbreviations:

GAPDH, glyceraldehyde-3-phosphate dehydrogenase; YWHAZ, tyrosine 3-monooxygenase/tryptophan 5-monooxygenase activation protein zeta; TNF, tumor necrosis factor; CCL2, C-C motif chemokine ligand 2; CXCL8, C-X-C motif chemokine ligand 8; IFNG, interferon gamma; IL1B, interleukin 1 beta; AGPAT1, 1-acylglycerol-3-phosphate O-acyltransferase 1; AGPAT2, 1-acylglycerol-3-phosphate O-acyltransferase 2; SREBF1, sterol regulatory element binding transcription factor 1; PPARG, peroxisome proliferator activated receptor gamma; FOXO1, forkhead box O1; FASN: Fatty acid synthase; SIRT1, sirtuin 1; SIRT3, sirtuin 3; PPARA, peroxisome proliferator activated receptor alpha; PRKAA2, protein kinase AMP-activated catalytic subunit alpha 2; PPARGC1A, PPARG coactivator 1 alpha; SOD2, superoxide dismutase 2; GPX1, glutathione peroxidase 1; PYGL, glycogen phosphorylase L; GSK3B, glycogen synthase kinase 3 beta; NFKB1, nuclear factor kappa B subunit 1; PTX3, pentraxin 3; MTTP, microsomal triglyceride transfer protein; APOB, apolipoprotein B; CD36, CD36 molecule;

SLC27A5, solute carrier family 27 member 5; SLC27A6, solute carrier family 27 member 6; PPIA, peptidylprolyl isomerase A; YWHAZ, tyrosine 3-monooxygenase/tryptophan 5-monooxygenase activation protein zeta; RPL19, ribosomal protein L19.

Disease Models & Mechanisms • Supplementary information

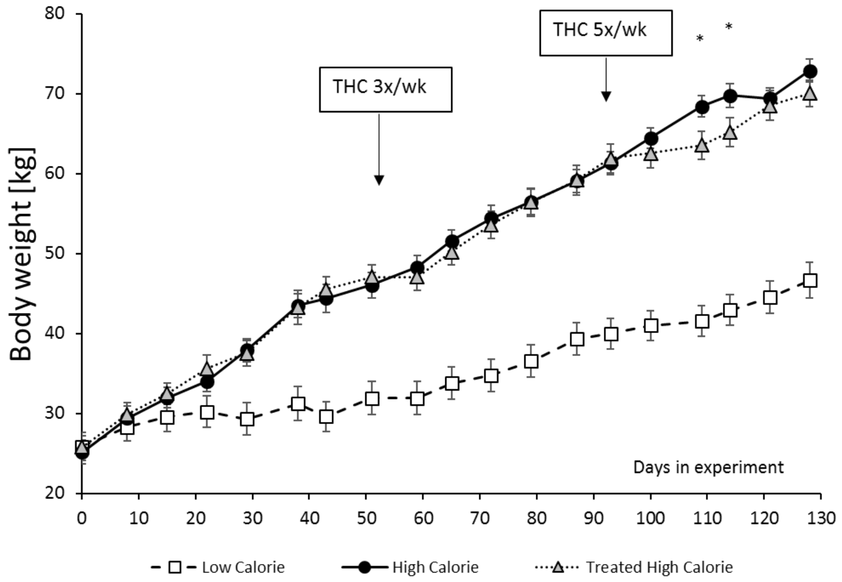

Figure S1. Body weight in the low calorie (LC), high calorie (HC) and thiamine-treated HC (THC) growing lambs. Repeated measures ANOVA analysis for HC vs. LC revealed an effect of the dietary treatment ($P < .0001$), time ($P < .0001$) and treatment x time interaction ($P < .0001$). Comparing HC with THC revealed an effect of time ($P < .0001$) and a treatment x time interaction ($P < .0001$) and a trend for thiamine treatment ($P = 0.09$). * Denotes $P < .05$ between HC and THC after Bonferroni-Holm correction.

Disease Models & Mechanisms • Supplementary information

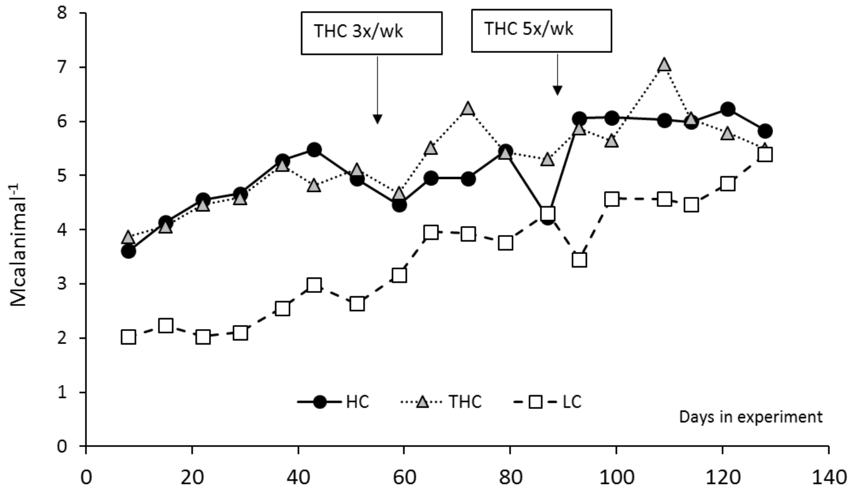

Figure S2. Daily group-average caloric intake in the low calorie (LC), high calorie (HC), and thiamine treated HC (THC) animals.

Disease Models & Mechanisms • Supplementary information

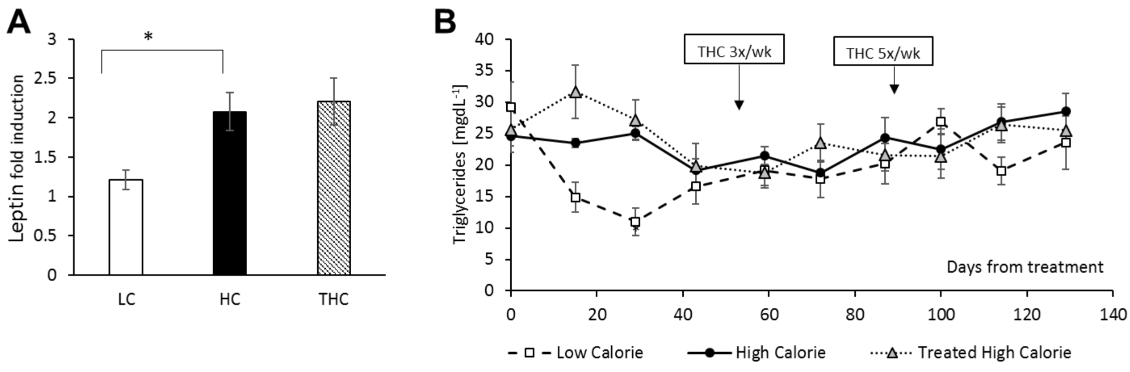

Figure S3. Plasma triglyceride and fasting leptin levels in the low calorie (LC), high calorie (HC) and thiamine-treated HC (THC) sheep. (**A**) Fasting plasma leptin concentration. One-way ANOVA detected an effect of the dietary treatment ($P = 0.02$). (**B**) Plasma triglycerides levels. Repeated-measures ANOVA analysis revealed an effect of the dietary treatment (HC vs. LC) ($P = 0.02$), time ($P = 0.001$), and treatment by time interaction ($P = 0.01$). * Denotes $P < 0.05$ by contrast t-tests.

Disease Models & Mechanisms • Supplementary information

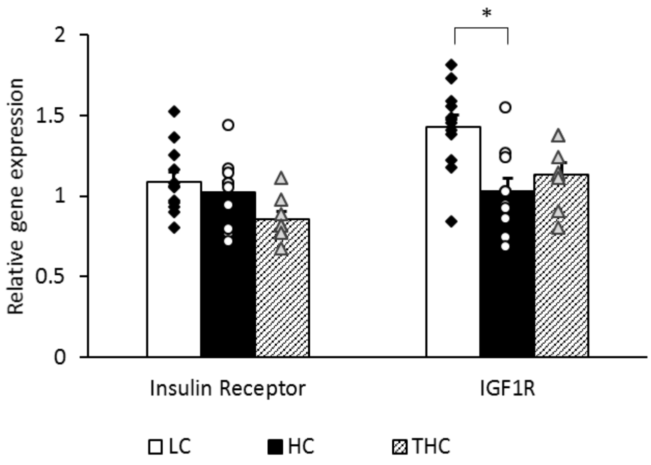

Figure S4. Comparative hepatic expression of genes involved in insulin sensitivity in the low-calorie (LC), high-calorie (HC), and thiamine-treated HC (THC) sheep. One way ANOVA detected an effect of treatment for Insulin receptor (P< 0.04) and *IGF1R* (P< 0.002). * Denotes P < 0.05 by contrast t-tests.

Abbreviations*:*

IGF1R, Insulin-like growth factor 1 receptor

Disease Models & Mechanisms • Supplementary information
